# Comparative Physiological and Proteomic Analysis Reveal Distinct Regulation of Peach Skin Quality Traits by Altitude

**DOI:** 10.3389/fpls.2016.01689

**Published:** 2016-11-10

**Authors:** Evangelos Karagiannis, Georgia Tanou, Martina Samiotaki, Michail Michailidis, Grigorios Diamantidis, Ioannis S. Minas, Athanassios Molassiotis

**Affiliations:** ^1^Laboratory of Pomology, Department of Agriculture, Aristotle University of ThessalonikiThessaloniki, Greece; ^2^Biomedical Sciences Research Center “Alexander Fleming”Vari, Greece; ^3^Laboratory of Agricultural Chemistry, Department of Agriculture, Aristotle University of ThessalonikiThessaloniki, Greece; ^4^Department of Horticulture and Landscape Architecture, Colorado State UniversityFort Collins, CO, USA; ^5^Western Colorado Research Center at Orchard Mesa, Colorado State UniversityGrand Junction, CO, USA

**Keywords:** antioxidants, peach [*Prunus persica* (L.) Batsch] fruit, proteins, elevation, ripening

## Abstract

The role of environment in fruit physiology has been established; however, knowledge regarding the effect of altitude in fruit quality traits is still lacking. Here, skin tissue quality characters were analyzed in peach fruit (cv. June Gold), harvested in 16 orchards located in low (71.5 m mean), or high (495 m mean) altitutes sites. Data indicated that soluble solids concentration and fruit firmness at commercial harvest stage were unaffected by alitute. Peach grown at high-altitude environment displayed higher levels of pigmentation and specific antioxidant-related activity in their skin at the commercial harvest stage. Skin extracts from distinct developmental stages and growing altitudes exhibited different antioxidant ability against DNA strand-scission. The effects of altitude on skin tissue were further studied using a proteomic approach. Protein expression analysis of the mature fruits depicted altered expression of 42 proteins that are mainly involved in the metabolic pathways of defense, primary metabolism, destination/storage and energy. The majority of these proteins were up-regulated at the low-altitude region. High-altitude environment increased the accumulation of several proteins, including chaperone ClpC, chaperone ClpB, pyruvate dehydrogenase E1, TCP domain class transcription factor, and lipoxygenase. We also discuss the altitude-affected protein variations, taking into account their potential role in peach ripening process. This study provides the first characterization of the peach skin proteome and helps to improve our understanding of peach's response to altitude.

## Introduction

During the ripening of peach fruit [*Prunus persica* (L.) Batsch], coordinated genetic and biochemical events occur that result in changes to texture, flavor, aroma, and color in both exocarp (skin) and mesocarp (flesh) tissues. Despite significant progress in illuminating the regulation of peach mesocarp ripening (Prinsi et al., 2011; Reig et al., 2015; Desnoues et al., 2016; Tadiello et al., 2016), knowledge regarding the metabolic shifts that underlie skin ripening is still lacking. Skin is of particular interest as this tissue is metabolically active during development and ripening, since it plays a central role in the synthesis of many compounds of interest (*e.g*., anthocyanins and aroma volatiles) (Tuan et al., 2015). The skin also constitutes a physical barrier between the external environment and the inner tissues, and its integrity is a key factor in preventing damage by physical injuries and pathogen attacks (Deytieux et al., 2007; Pilati et al., 2014).

Environmental factors, such as night/day temperature and wavelengths of ambient light or even a combination between all these parameters, can uncouple the processes of peach ripening (Reig et al., 2015). The majority of these factors are firmly associated with altitude; thus, altitude is one of the most crucial parameters that are involved in the final quality of fruits (Ziogas et al., 2010). Despite the fact that studies on the impact of environment in fruit skin biology will broaden our knowledge in fruit ripening, the role of environmental stimuli, such as altitude is yet to be understood (Leida et al., 2012).

Given the complexity of the ripening process, the use of post genomic tools that allow the global evaluation of the molecular processes triggered within the fruit is particularly important (Molassiotis et al., 2013). Metabolic studies on peach fruit ripening showed that amino acid levels decreased, coupled with the elevation of transcripts involved with amino acid and organic acid catabolism during ripening stage, consistent with the mobilization of amino acids to support respiration (Lombardo et al., 2011). Microarray transcriptome profiling identified several putative peach ripening-related genes belonging to several families including MADS-box, AUX/IAA, bZIP, bHLH, Myb (Trainotti et al., 2006), ethylene biosynthesis/signaling (*ACS1, ACO1/ETR1, ETR2*), and cell wall metabolism (*PG* and *EXP2*) (Ziliotto et al., 2008; Wang et al., 2013). Recently, it was also proposed that CTG134 gene, a precursor of a peptide hormone of the RGF/GLV, regulates ethylene-auxin cross-talk during peach fruit ripening (Tadiello et al., 2016). Given that the proteome is closer to the fruit phenotype than is the genome or the transcriptome along with the fact that it is more directly responsive to environmental stimuli (Chan, 2012), proteomic analysis provides a comprehensive view of fruit's adaptation to different environments. Almost all past proteomic studies of peach fruit were focused on mesocarp tissue (Lara et al., 2009; Nilo et al., 2010, 2012; Hu et al., 2011; Prinsi et al., 2011; Zhang et al., 2011; Giraldo et al., 2012); whereas research concerning skin proeome as an isolated component during peach ripening have not been reported yet.

On the basis of the evidence summarized above, we hypothesized that the altitude would significantly influence the ripening behavior of the peach fruit skin tissue. Thus, the first step of the present work was designed to characterize the impact of altitude on peach skin physiology. We show that high altitude regulates the pigmentation and the antioxidant dymanic of skin tissue. At the second step of analysis, two-dimensional gel electrophoresis (2-DE-PAGE) together with liquid chromatography-tandem mass spectrometry (LC-MS/MS), were employed to study the changes in protein profiles between low and high altitude orchards. The physiological and biochemical implications of these skin proteins were discussed in the context of peach fruit ripening sydrome.

## Materials and methods

### Fruit material, sampling strategy, and quality traits

In order to investigate the effect of altitude in peach fruit quality, skin tissue samples of the cultivar (cv) “June Gold” were collected at commercial harvest from 16 independently managed orchards at 2012 year in two regions with significant difference in altitude. The first group of 8 orchards was located at Meliki (Imathia, North Greece) at about 71.5 m (mean) altitude (defined as low altitude), and the second group of 8 orchards located at Velvedos (Kozani, North Greece) at about 495.7 m (mean) altitude (defined as high altitude). During the ripening period, the climate data (day/night temperature and relative humidity) of the two altitude regions were recorded (Supplementary Figure S1). All orchards consisted of 6–8-year old trees, planted at 5 × 5 m spacing between rows and along the row, grafted onto GF-677 rootstock, trained in open vase and subjected to standard cultural practices for peach production. Fruit samples were harvested and analyzed immediately after harvest and subsequently skin and flesh tissues were frozen with liquid nitrogen and stored at −80°C for further analysis.

Peach fruit were harvested from each orchard at the commercial mature pre-climacteric stage, based on ground color and fruit firmness and divided into 8 lots (orchards) of 40 fruits for each growing altitude. Harvest quality evaluation included subjunctive and objective analysis of skin color, and objective analysis of fruit firmness, soluble solids concentration (SSC), and titratable acidity (TA). Skin foreground (darkest red) and background color on two opposite sides of each fruit were measured objectively using a colorimeter (Konica Minolta CR200 Chroma Meter, Konica Minolta Sensing, Inc., Osaka, Japan) and the CIE (Commission Internationale de l'Eclairage) parameters (L^*^, a^*^, b^*^), as previously described (Goulas et al., 2015). The percentage of red-blushed surface was subjectively estimated as the percentage of red overcolor on the total peach surface. Fruit firmness (kg), titratable acidity (TA, malic acid, %) and soluble solids content (SSC, %) of the peaches were measured according to procedure described in detail elsewhere (Minas et al., 2016). Furthermore, skin samples from two selected orchards (7 years old trees with GF-677 as rootstock) from low and one from high altitude region (situated 50 and 550 m above sea level; herein named as low- and high-altitude reference orchards, respectively) were used for both DNA nicking and proteomic analysis.

### Anthocyanins and carotenoids (β–Carotene equivalent) determinations

Peach outer pericarp (skin) anthocyanins at commercial harvest stage were extracted with 80% ethanol + 1% HCl, as described by Giusti and Wrolstad (2005). The contend of anthocyanins was determined by the pH differential method (Wolfe et al., 2003) and results were expressed as μg g^−1^ fresh weight (FW). β-Carotene extracts were prepared using 1 or 2.5 g (skin or flesh) by adding hexan: Acetone: Ethanol (50:25:25 v/v) and incubation for 24 h at 5°C in darkness. β-Carotene concentration was calculated in both flesh and skin tissues through the absorbance at 450 nm (Kuti, 2004).

### Total phenols, flavonoids, and total antioxidants assays

Flesh and skin tissues at commercial harvest stage were extracted following conditions previously described (Asami et al., 2003). Total phenols content was determined by the reaction of the extract with the Folin-Ciocalteu reagent (Asami et al., 2003) and results were expressed as μg gallic acid equivalent (GAE) g^−1^ FW. Total flavonoids concentration was assayed in skin tissues as reported by Cvek et al. (2007), using catehin as standard and results expressed as μg catehin g^−1^ FW. Total antioxidant activity was determined in flesh and skin tissues using three different methodologies. The ferric reducing antioxidant power (FRAP) assay was performed according to Benzie and Strain (1996). The antioxidant potential of the extracts was determined from a standard curve using trolox as equivalent and expressed as μg Trolox g^−1^ FW. The 2,2′-azino-bis-3-ethylbenzthiazoline-6-sulphonic acid (ABTS) free radical scavenging activity of sample was evaluated as reported by Re et al. (1999). Aqueous solution of trolox was used for the calibration curve and the results were expressed as μg Trolox g^−1^ FW. The 1,1-diphenyl-2-picrylhydrazyl (DPPH) assay was determined according to Brand-Williams et al. (1995). An aqueous solution of Trolox was used for the calibration curve and the results were expressed as μg Trolox g^−1^ FW.

### DNA protection assay

The ability of skin extracts to protect the plasmid DNA from the destructive effects of hydroxyl radicals (•OH) was assessed by the DNA nicking assay described by Hu and Kitts (2001). For evaluating •OH-induced oxidative breakage generated by Fenton's reagent, samples from a reference low- and high-altitude orchards (with 50 and 550 m altitude, respectively) were collected at four distinct developmental stages (S2, green; S3, yellow-green; S4I, commercial harvest/pre-climacteric phase; S4II, tree-ripe/climacteric phase) based on the double sigmoidal fruit growth curve (data not shown). The reaction mixture contained 0.5 μg of supercoiled pBR322 plasmid DNA, 12 μL of Fenton's reagent (16.2 mM H_2_O_2_, 3.6 mM ascorbic acid and 36 mM FeCl_3_) followed by the addition of skin phenolic extracts (100 μM GAE) and the final volume of the mixture was brought up to 20 μL using double-distilled water. The reaction mixtures were allowed to incubate for 30 min at 37°C. Agarose gel electrophoresis and ethidium bromide staining was conducted according to Ziogas et al. (2010). The supercoiled (SC), open circular (NC; after a single-strand break), and linear (NL; after double-strand break) forms of plasmid DNA were visualized using UV transilluminator system. The Image J software (National Institutes of Health, NIH) was used to quantify DNA strand breaks compared to the intensity of the supercoiled DNA. The assay was run in triplicate and averaged.

### Two-dimensional gel electrophoresis, protein visualization, and image analysis

Skin tissue samples from reference low- and high-altitude orchards (with 50 and 550 m altitude, respectively) at the commercial harvest stage were ground in liquid nitrogen with a pestle and mortar and soluble skin proteins were extracted through a phenol-based extraction protocol. Total proteins were extracted from 4 g of each homogenized sample with 5 mL extraction buffer containing 100 mM Tris-HCl pH 8.8, 20 mM DTT, 1 mM PMSF, 0.5% Triton X-100, 5 mM EDTA, 30% saccharoze, and Complete Mini protease inhibitor cocktail tablet (Roche Molecular Biochemicals) (Tanou et al., 2009). An equal volume of phenol-Tris saturated was added and the protein extracts were stirred for 20 min at 4°C and centrifuged at 15000 g for 10 min at 4°C. Following the phenolic phase collection, a 5:1 cold 0.1 M ammonium acetate (dissolved in methanol) was added and the samples kept at −20°C overnight. After centrifugation (10000 g, 10 min, 4°C), the precipitated protein pellet was washed one time with ice-cold ammonium acetate, three times with ice-cold 70% acetone and subsequently was dried at a Thermoblock (Thermo Block 780). The proteins were resolubilized in rehydration buffer containing 42% Urea (w/v), 15.2% Thiourea (w/v), 4% CHAPS (w/v), 0.5% Triton X-100 (v/v), 0.3% DTT (w/v), and 0.5% ampholyte (v/v). Protein concentrations were measured according to Bradford (1976), using a Bio-Rad protein assay kit (Kit II, cat. n. 500–0002). Bovine serum albumin (Sigma) was used as a standard.

Samples' aliquots containing 50 μg proteins were loaded onto 11 cm pH 3–10 non-linear IPG strips, and isoelectric focusing was performed on a PROTEAN IEF Cell system (BIO-RAD) for a total of 35000 Vh at 20°C. Subsequently, the strips were equilibrated with 1.11% (w/v) DTT and afterwards with 4% (w/v) iodoacetamide in equilibration buffer containing Urea (36%), SDS (2%), 1.5 M Tris-HCl pH 8.8 (3.3%), and Glycerol (30%). Following equilibration, the strips were loaded on 12.5% Tris—HCl 1.0 mm Criterion™ Precast (BIO-RAD) gels on a Criterion Dodeca™ Cell (BIO-RAD) device. Following the procedure of silver nitrate staining (Tanou et al., 2010), 2DE-gels were scanned with Bio-Rad GS-800 Calibrated Densitometer equipped with PDQuest Advanced 2-D Gel Analysis Software. Statistical analysis was done by one-way analysis of variance significance (*P* ≤ 0.05) and individual means were compared using student's *t*-test (significance level 95%). The statistical significant differences further combined by the quantitative 1.5 fold change of spot volume (Supplementary Table S3). At least three biological replicates were performed for each treatment.

### LC–MS/MS analysis

Selected gel spots, with differential intensity, were subjected to tryptic in-gel digestion and analyzed by LC-MS/MS using a LTQ Orbitrap XL Mass spectrometer (Thermo Fisher Scientific, Bremen, Germany) coupled online with a nanoLC Ultimate 3000 chromatography system (Dionex, Sunnyvale, CA), as described in detail by Ainalidou et al. (2016). Raw files were searched against the Uniprot *Prunus persica* protein database (downloaded 21/1/2014) containing 28650 protein sequences using the SequestHT software. Protein identification required minimal XCorr values of 2.0, and 2.5 for charge states of doubly, and triply precursor ions. To validate protein identification with one single peptide (spot 8033 at Supplementary Table S3) additional information are provided in Supplementary Figure S3.

### Bioinformatics analysis

To obtain additional protein information for subsequent functional validation, all of the differentially expressed proteins were subjected to a global protein network analysis using STRING tool (version 10) (http://string.embl.de/) (Szklarczyk et al., 2011). This tool was applied to predict protein—protein interactions based on both physical and functional associations by querying the list of proteins through multiple resources, including (a) experimentally confirmed interactions, (b) pathway knowledge from curate databases, (c) automated text-mining based on Medline/PubMed abstract and full-text articles, (d) predicted interactions using genomic information and co-expression analysis, and (e) interactions that were observed in one organism and then transferred to others (Szklarczyk et al., 2011). Since protein identification was based upon different organisms listed in the National Center for Biotechnology Information (NCBI) Viridiplantae database, all identified peach proteins were blasted against the *Arabidopsis thaliana* TAIR10 (The Arabidopsis Information Resource) protein database (http://www.arabidopsis.org/) with the intention of obtaining annotated protein entries for PPI tools. Results with the highest score and lowest E value were considered as relevant for each identified peach protein (Supplementary Table S2).

### Statistical analysis

For the physiological data, Student's *t*-test (using three replicates) at 5% level of significance was prepared by using the statistical package SPSS 12.0 (SPSS Inc., Chicago, USA). Pearson's correlation coefficients and principal component analysis (PCA) were performed to obtain an overview of correlation among fruit quality traits and protein function categories.

## Results and discussion

### The phenotype of peach skin is remarkably affected by altitude

**Figure 1:**
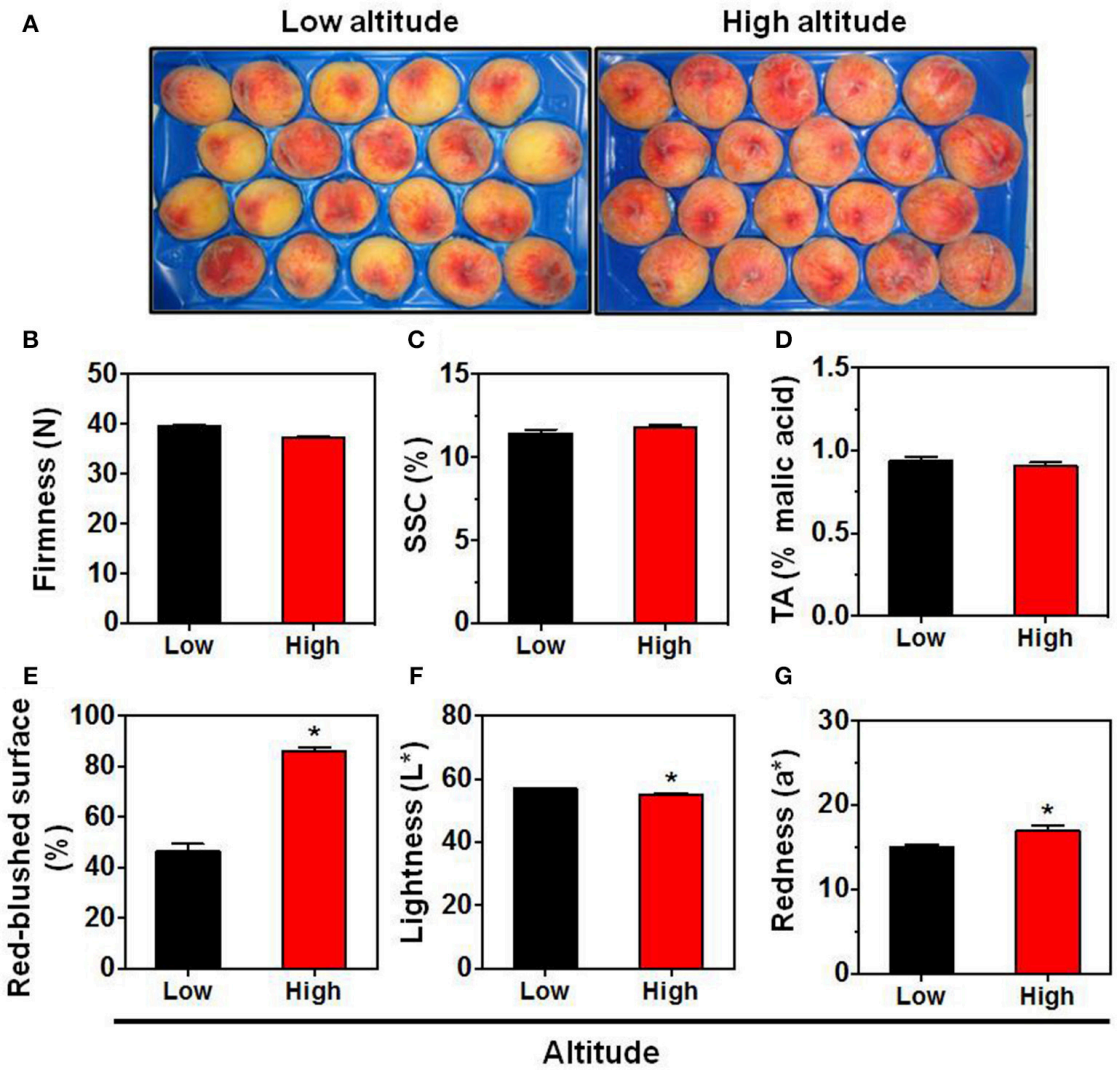
**Phenotype (A)**, firmness **(B)**, soluble solids concentration (SSC) **(C)**, titratable acidity (TA) **(D)**, red overcolor coverage **(E)**, skin lightness (L^*^) **(F)**, and skin redness (a^*^) **(G)** of “June Gold” peaches. Fruit were collected at commercial harvest stage from 16 independently orchards that located in two regions with low altitude (8 orchards; mean 71.5 m) and high altitude (mean 495.7 m). For each orchard, the fruit were divided into 8 replicates, each with 5 fruit. The values shown are the mean ± *SD*. Bars with asterisk are significantly different at *P* = 0.05 (Student's *t*-test).

Peach fruit, particularly skin tissue, undergo a broad metabolic reprogramming and definitive specialization during fruit ripening that involves internal signals refined by environmental cues (Frett et al., 2014). Nonetheless, no previous work has determined the impact of environmental factors in peach skin biology. Herein, we characterize the relationship between altitude and peach fruit quality and physiology using various parameters. Peach sampled at commercial harvest stage from sixteen orchards located at two environmental conditions had similar firmness (Figure 1B), SSC (Figure 1C), and TA (Figure 1D), which are considered suitable indicators of the peach ripening status (Iglesias and Echeverría, 2009). This indicates that at this stage both environmental-based types of peach fruit were at the same physiological status and the various quality differences can be mainly attributed to the environment without alterations owing to adequate ripening stage. From the presented phenotypical data it was clear that peach fruit altered their physiognomy in response to the two different regions (Figure 1A). The first physiological response was the modulation of skin color parameters, such as lightness (L^*^) and redness (a^*^) (Figures 1F,G). In support, the percentage (%) of red-blushed surface of peach skin was almost doubled (84.5%) in the high altitude environment (Figure 1E), which agrees with the notion that the red coloration in other fruit species is stimulated by the growing altitude (Espley et al., 2007).

### High-altitude environment induce antioxidant activity and pigmentation in peach fruit skin

**Figure 2:**
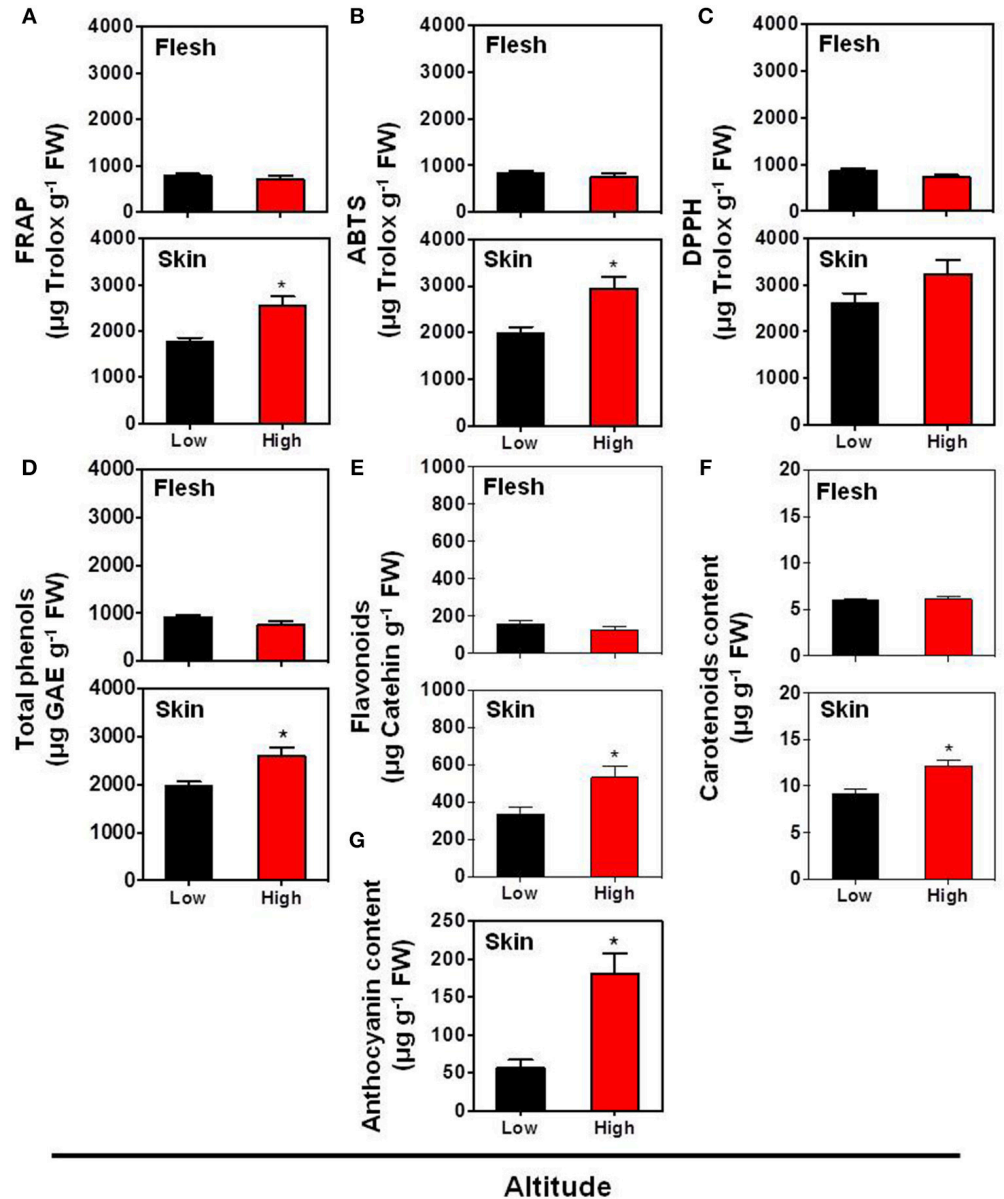
**Impact of altitude on the antioxidant capacity according to FRAP assay (A)**, ABTS assay **(B)**, DPPH assay **(C)**, as well as on the contents of total phenols **(D)**, flavonoids **(E)**, carotenoid **(F)**, and anthocyanin **(G)** in the skin and flesh tissues of “June Gold” peaches. Additional experimental details as described in Figure 1. The values shown are the mean ± *SD*. Bars with asterisk are significantly different at *P* = 0.05 (Student's *t*-test). Data are means of values obtained from eight biological replicates, each with 5 fruits.

Previous studies indicated that the fruit skin pigmentation is linked with the biosynthesis of health promoting bioactive substances (Butelli et al., 2008). In the present study, the total antioxidant activity, assessed with three different assays (FRAP, ABTS, and DPPH assays) (Figures 2A–C) together with the total phenols content (Figure 2D), were not significantly differed in the flesh tissue between the low and high altitude areas. The same trend was also described by Montevecchi et al. (2012) in various peach cultivars grown at difference landraces. However, the antioxidant capacity (evaluated by FRAP and ABTS assays) and the total phenols content were induced in skin tissue by the high altitude (Figures 2A,B,D). Another interesting finding in this work is the activation of pigments biosynthesis by high altitude in the skin tissue, as evidenced by the accumulation of carotenoids (β-carotene equivalent) (Figure 2F), total flavonoids (Figure 2E) and particularly anthocyanins (Figure 2G), suggesting that environmental factors at a higher altitude, such as low temperature (Supplementary Figure S1), favored pigments biogenesis in peach fruit, as already proposed in apple (Espley et al., 2007; Lin-Wang et al., 2011). All these differences between the two growing regions could be a result of specific modifications at ambient light, UV radiation, day length, temperature difference between day and night, or even a combination between all these parameters that affect fruit physiology. Thus, further research is needed to unravel the specific function of these climatic factors in peach fruit quality.

### Interaction between fruit development stage and altitude provides different perspectives on peach skin-derived DNA oxidation properties

**Figure 3:**
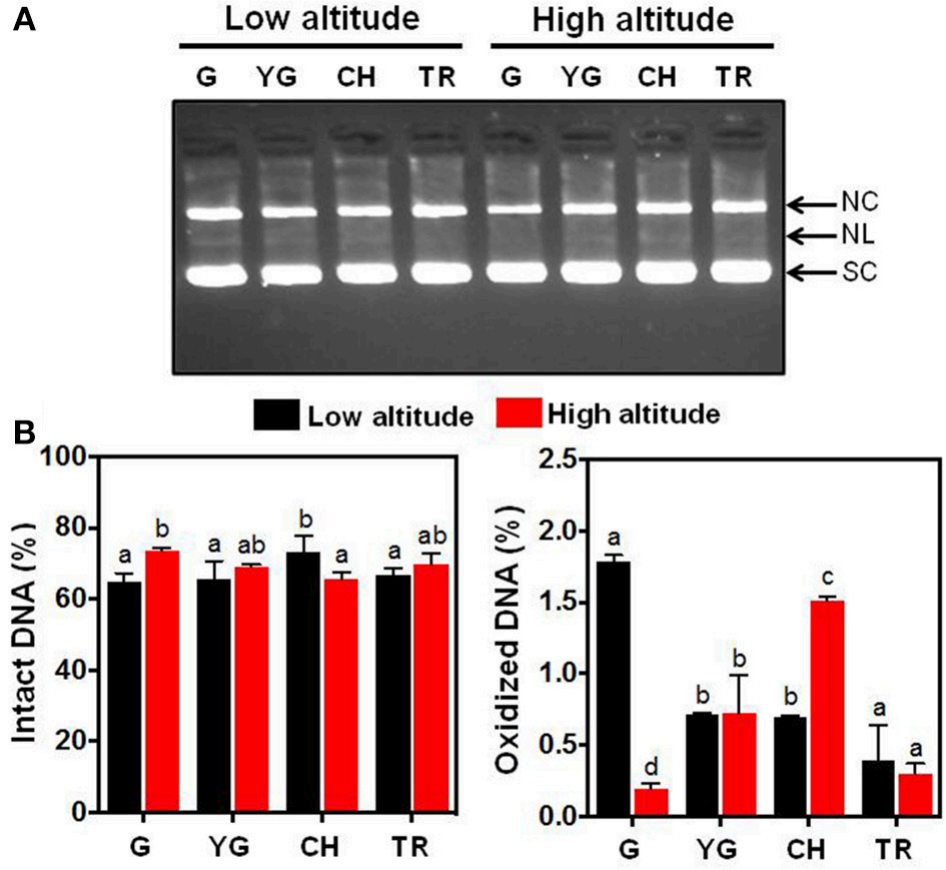
**(A)** Inhibitory effect of the fruit skin-extracted phenols from “June Gold” peaches grown at two reference orchards located in two regions differing in altitude (50 and 550 m) across four stages of fruit development (green, G; yellow green, YG; commercial harvest, CH; tree ripe, TR) in preventing pBR322 DNA nicking caused by •OH generated from a Fenton-reaction mixture (Fe^3+^-EDTA/ascorbic acid/H_2_O_2_). SC, supercoiled DNA; NC, open circular DNA (after a single-strand break); NL, linear DNA (after double-strand break). **(B)** Densitometric quantification of the intensity (%) of intact (SC fraction) and oxidized (NC and NL fractions) DNA. Data represent mean ± *SD* (n = 3). Values followed by the same letter are not significantly different at *P* = 0.05.

The substantial differences in antioxidant behavior observed in fruit skin of the two altitudes at harvest (Figures 2A,B) prompted us to study the relative protective effect of skin phenolic extracts at different developmental stages and altitudes on DNA strand cleavage by •OH (Figure 3). DNA nicking in-gel assay (Figure 3A) and quantitative analysis (Figure 3B) of intact and oxidized DNA showed that no differences exist in the ability of skin extracts from the two altitudes to protect DNA from oxidation at yellow green (YG) and tree ripe (TR) stages. Low altitude-derived skin extracts in the green stage (G) had lower ability to protect DNA from breakage (Figure 3B), as visualized by the accumulation of open circular (NC) and linear (NL) forms, compared with the corresponding samples from the high altitude (Figure 3A). On the other hand, it was observed that commercial harvest (CH) stage-extracted phenols from low altitude exhibited relative higher protection against •OH-induced DNA damage (Figures 3A,B), providing proof that the ripening stage and the altitude had a significant role in the production of phytochemicals responsible for DNA protection. It is noteworthy that there was no strict correlation between the ability of phenolic extracts to inhibit DNA damage (Figures 3A,B) and the phenols content (Figure 2D), an observation that was also noted in olive fruit by Ziogas et al. (2010).

### Identification of peach skin proteins affected by low- and high-altitude environment

**Figure 4:**
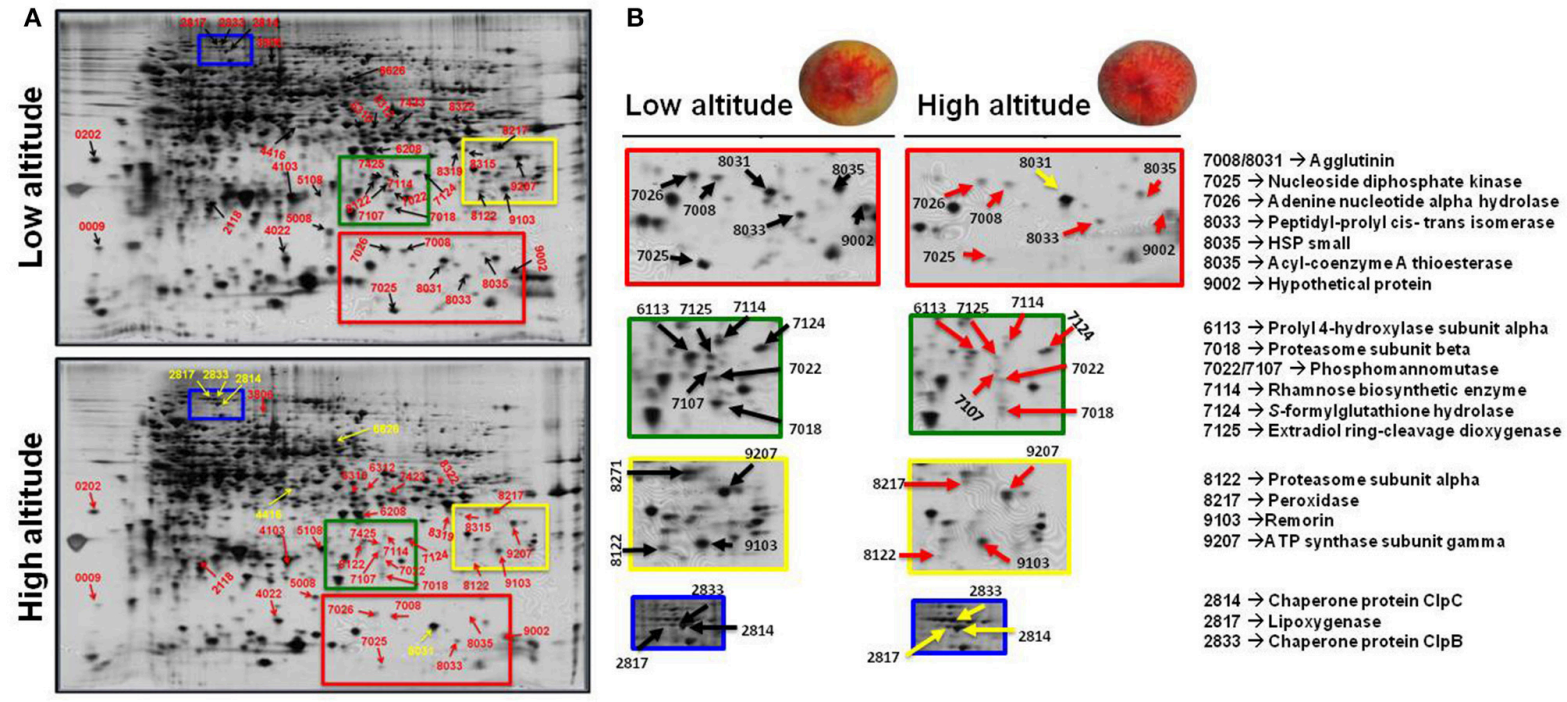
**The impact of altitude on the proteome of peach skin tissue at commercial harvest stage**. “June Gold” peaches grown at two reference orchards located in two regions differing in altitude (50 and 550 m). **(A)** Profile of the 2DE-PAGE patterns of peach fruit harvested at low- and high-altitude environments. The numbers indicate differentially expressed proteins and correspond to the numbers listed in Table 1. Up- and down-regulated proteins are presented with yellow and red arrows, respectively. **(B)** Representative protein spots and corresponding proteins showing quantitative differences between the two experimental sites.

**Figure 5:**
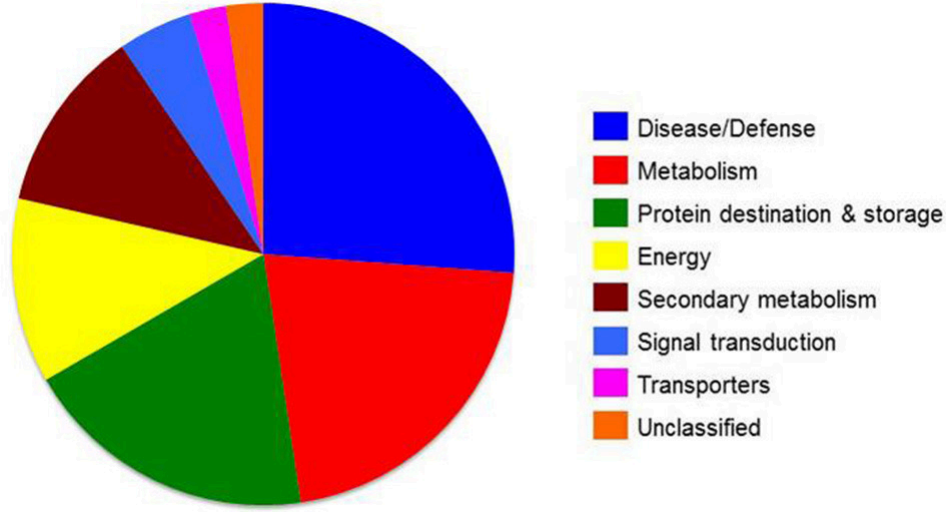
**Functional groups of differentially expressed skin proteins identified at commercial harvest stage from “June Gold” peaches grown at two reference orchards located in two regions differing in altitude (50 and 550 m)**. Proteins were classified into functional classes according to Bevan et al. (1998).

To analyze the cellular basis underpinning the phenotypes observed in peach fruit that cultivated in the different environments (Figure 1A), a proteomic analysis of “June Gold” peach skin samples at commercial harvest stage was performed. Through 2-DE analysis, 850 spots were detected, 38 of which were modified under the different environmental conditions based on the Student's *t*-test and further validated by the 1.5 fold threshold change (Supplementary Table S1). Those spots were excised; trypsin digested, and analyzed by nano-LC–MS/MS. Representative spots are magnified and labeled in Figures 4A,B. LC/MS-MS analysis identified 42 proteins that were sorted into eight functional classes according to Bevan et al. (1998). The basic information of the identified proteins are listed in Table 1 (detailed information are provided at Supplementary Table S3), such as protein name, score, molecular weight, pI, unique peptides. Identified proteins in more than one protein spots suggest that a small number of different spots expressed or undergo post-translation modifications. Such proteins were adenine nucleotide hydrolase (two protein spots), agglutinin (two protein spots), and phosphomannomutase (two protein spots). The accumulation of many proteins has been changed (increased or decreased) in response to different altitudes (Table 1). Differently expressed proteins belonged to different metabolic pathways, including 11 proteins associated with defense, followed by main/primary metabolism (9 proteins), destination and storage (8 proteins), energy (5 proteins), secondary metabolism (5 proteins), signal transduction (2 proteins), H^+^ transporters (1 protein), and one protein with unknown biological function (Figure 5). The identified proteins were detected in nine (9) different parts of the cell, including chloroplast (50.0%), cytoplasm (21.4%), mitochondrion (7.1%), cytosol (7.1%), cell wall (4.8%), vacuole (2.4%), nucleus (2.4%), endoplasmic reticulum (2.4%), and peroxisome (2.4%) (Supplementary Figure S2).

**Table 1:** **List of peach (cv. “June Gold”) skin proteins at commercial harvest stage identified by LC-MS/MS in fruits cultivated in the low- and high-altitude reference orchards**.

**Spot No.^a^**	**Protein name**	**Acc. No.^b^**	**U/D^c^**	**Functional categories^d^**	**Coverage^e^ (%)**	**Score^f^**	**MW [kDa]^g^**	**Calc. pI^h^**	**Unique Peptides No.^i^**
9	Elicitor-responsive protein	M5X0Z3	D	10.99-Signal Transduction/Others	15.43	16.80	19.1	4.27	2
202	Plastid-lipid associated protein PAP/fibrillin family protein isoform	M5WMK2	D	11.05-Disease/Defense/Stress responses	10.20	7.49	37.8	4.81	3
2118	GDSL esterase/lipase CPRD49 isoform	M5XRT2	D	01.06-Metabolism/Lipid and sterol	20.31	11.37	28.6	5.45	4
2814	Chaperone protein ClpC	M5WCY0	U	06.01-Protein destination and storage/Folding and stability	13.90	58.80	102.0	6.73	11
2817	Lipoxygenase	M5X5W8	U	01.06-Metabolism/Lipid and sterol	27.50	53.67	89.8	5.62	15
2833	Chaperone protein ClpB	M5WSD5	U	06.01-Protein destination and storage/Folding and stability	6.83	19.34	110.3	6.51	7
3806	NADH dehydrogenase [ubiquinone] iron-sulfur protein	M5WWP9	D	02.20-Energy/Electron-transport	25.41	68.07	85.8	6.83	15
4022	Adenine nucleotide alpha hydrolase	M5VKC1	D	11.05-Disease/Defense/Stress responses	20.11	7.31	19.3	6.29	3
4103	Ascorbate peroxidase	M5VZU3	D	11.06-Disease/Defense/Detoxification	65.20	71.83	27.3	6.16	10
4416	Pyruvate dehydrogenase E1 component subunit alpha	M5XCJ6	U	02.30-Energy/Photosynthesis	22.45	30.41	47.6	7.02	7
5008	Glutathione peroxidase	M5WAJ5	D	11.06-Disease/Defense/Detoxification	8.86	5.76	25.9	9.09	2
5108	6-phosphogluconolactonase	M5X1U6	D	02.07-Energy/Pentose phosphate	6.71	12.43	34.1	8.75	2
6113	Prolyl 4-hydroxylase subunit alpha	M5XS77	D	20.99-Secondary metabolism/Others	7.07	7.60	33.0	6.71	2
6208	Annexin	M5W098	D	11.05-Disease/Defense/Stress responses	18.99	17.88	35.9	6.64	5
6310	HSP20	M5XEL2	D	06.01-Protein destination and storage/Folding and stability	9.12	10.76	33.8	6.46	3
6312	Sinapyl alcohol dehydrogenase	M5WBG4	D	20.1-Secondary metabolism/Phenylpropanoids/Phenolics	36.74	100.43	38.9	6.76	11
6626	TCP domain class transcription factor	M5X5S1	U	06.01-Protein destination and storage/Folding and stability	28.22	86.43	59.2	6.49	13
7008	Agglutinin	M5WR60	D	11.02-Disease/Defense /Defense-related	54.09	35.22	17.9	6.34	6
7018	Proteasome subunit beta	M5XCF1	D	06.13-Protein destination and storage/Proteolysis	51.47	90.69	29.1	5.85	8
7018	Glutathione S-transferase	M5WTZ4	D	11.06-Disease/Defense/Detoxification	41.12	30.88	24.7	6.30	6
7022	Phosphomannomutase	M4QFW7	D	01.05-Metabolism/Sugars and polysaccharides	21.05	50.55	28.1	6.80	5
7025	Nucleoside diphosphate kinase	M5X104	D	01.03-Metabolism/Nucleotides	70.95	101.37	16.5	6.95	8
7026	Adenine nucleotide alpha hydrolase	M5W0J9	D	11.05-Disease /Defense/Stress responses	40.74	15.02	17.8	6.68	5
7107	Phosphomannomutase	M4QFW7	D	01.05-Metabolism/Sugars and polysaccharides	16.19	32.05	28.1	6.80	4
7114	Rhamnose biosynthetic enzyme	M5WBB7	D	01.05-Metabolism/Sugars and polysaccharides	24.08	30.62	33.4	6.57	6
7114	Electron transfer flavoprotein subunit beta	M5XZZ7	D	02.20-Energy/Electron-transport	17.13	29.74	27.5	6.61	6
7124	S-formylglutathione hydrolase	M5W9G4	D	11.06-Disease /Defense/Detoxification	32.06	47.28	32.2	7.08	7
7125	Extradiol ring-cleavage dioxygenase	M5WGS1	D	20.99-Secondary metabolism/Others	31.02	29.07	30.1	6.86	7
7423	Cinnamyl alcohol dehydrogenase	M5WB20	D	20.1-Secondary metabolism/Phenylpropanoids/Phenolics	49.30	61.70	38.4	6.93	12
8031	Agglutinin	M5WNR2	U	11.02-Disease/Defense/Defense-related	69.62	142.10	17.9	7.49	7
8033	Peptidyl-prolyl cis-trans isomerase	M5W6L5	D	06.01-Protein destination and storage/Folding and stability	8.09	2.19	18.2	8.47	1
8035	HSP small	M5WDA5	D	06.01-Protein destination and storage/Folding and stability	33.54	34.49	18.1	8.32	3
8035	Acyl-coenzyme A thioesterase	M5WHT9	D	20.1-Secondary metabolism/Phenylpropanoids/Phenolics	23.13	22.41	17.1	8.09	4
8122	Proteasome subunit alpha	M5VQX2	D	06.13-Protein destination and storage/Proteolysis	32.93	17.84	27.1	7.96	7
8217	Peroxidase	M5W030	D	11.06-Disease/Defense/Detoxification	30.09	67.22	35.6	7.96	8
8217	Uricase	M5WGE6	D	01.99-Metabolism/Others	20.52	44.60	34.8	8.06	6
8315	c-1-tetrahydrofolate synthase	M5VKQ0	D	01.01-Metabolism/Amino acid	14.72	15.51	31.8	7.55	6
8319	Fructose-bisphosphate aldolase	M5W2H9	D	02.30-Energy/Photosynthesis	18.16	28.53	38.4	7.36	6
8322	Class I glutamine amidotransferase	M5WGA9	D	01.01-Metabolism/Amino acid	26.24	75.61	47.2	8.68	13
9002	Hypothetical protein	M5W0H8	D	13 Unclassified	33.13	38.04	18.1	8.29	4
9103	Remorin	M5XKT5	D	10.04-Signal transduction	45.18	56.89	21.8	8.02	10
9207	ATP synthase subunit gamma	M5WB68	D	07.22-Transporters/Transport ATPases	37.77	114.33	35.2	9.04	10

a *Spot No, protein spot number on the reference gel maps presented in Figure 4A*;

b *Acc. No, Uniprot accession number*;

c *U/D, (U; up-regulated protein, D; down-regulated) protein for comparison purposes, low altitude samples served as control*;

d *Functional category, proteins ontologically classified into functional categories proposed by of Bevan et al. (1998)*;

e *Coverage; percentage of sequence coverage obtained with identified peptides with SEQUEST software for the orthologous protein*;

f *Score; SEQUEST score; the sum of all peptide Xcorr values above the specified score threshold*;

g *MW, molecular weight*;

h *Calc. pI, theoretical isoelectric point*;

i *Uni. Pept. No, Number of unique identified peptides*.

### Characterization of quality traits and protein changes by principal component analysis

**Figure 6:**
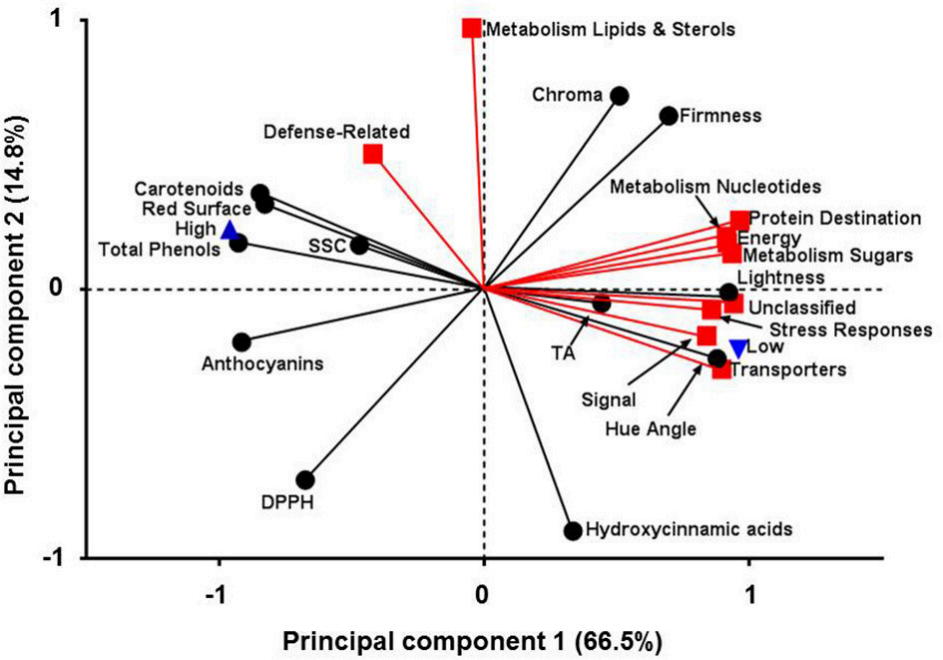
**Principal components analysis for fruit quality attributes and protein function categories in peach fruit cultivated in different altitude**. First principal component (66.5%) on x-axis and second principal component (14.8%) on y-axis.

Principal component analysis has been previously used to monitor the quality characteristic of peach fruit (Cantín et al., 2010). In this work, PCA was applied in order to determine the possible relationships between quality and proteomic data in response to different environmental conditions (Figure 6). It is evident that the altitude affected several parameters that were studied in the current experiment. Particularly, three groups of variables (Group 1; total phenols, FRAP, ABTS, flavonoids, Group 2; proteins related to sugars/amino acids metabolism and secondary metabolism, Group 3; proteins involved in energy and detoxification) that displayed correlation coefficients above 0.99 were presented by the first part of each group based on Pearson's correlation coefficient (Supplementary Table S4). PCA analysis of low and high altitude was demonstrated by two interpretable factors that described about 79.1% of the total variation in the samples (61.3% for PC1 and 17.8% for PC2) (Figure 6). PC1 was more closely linked to the most of the protein functions as well as to color and to antioxidant-related traits; the first two were positively connected while the third negatively. PC2 was strongly associated with proteins that involved in the metabolism of lipids and sterols showing higher value inversely to hydroxycinnamic acids. Altitude was distributed on PC1, in which the negative values were represented by an increase in antioxidant-related parameters at high altitude. On the other hand, low altitude was positively associated with the induction of specific protein categories, such as signal, transporters and stress response, as well as with fruit color features, such as Hue angle and lightness (Figure 6). To our knowledge this is the first study that relates proteomic features of peach fruit tissue with its quality characteristics and response to altitude.

### Global protein network analysis

**Figure 7:**
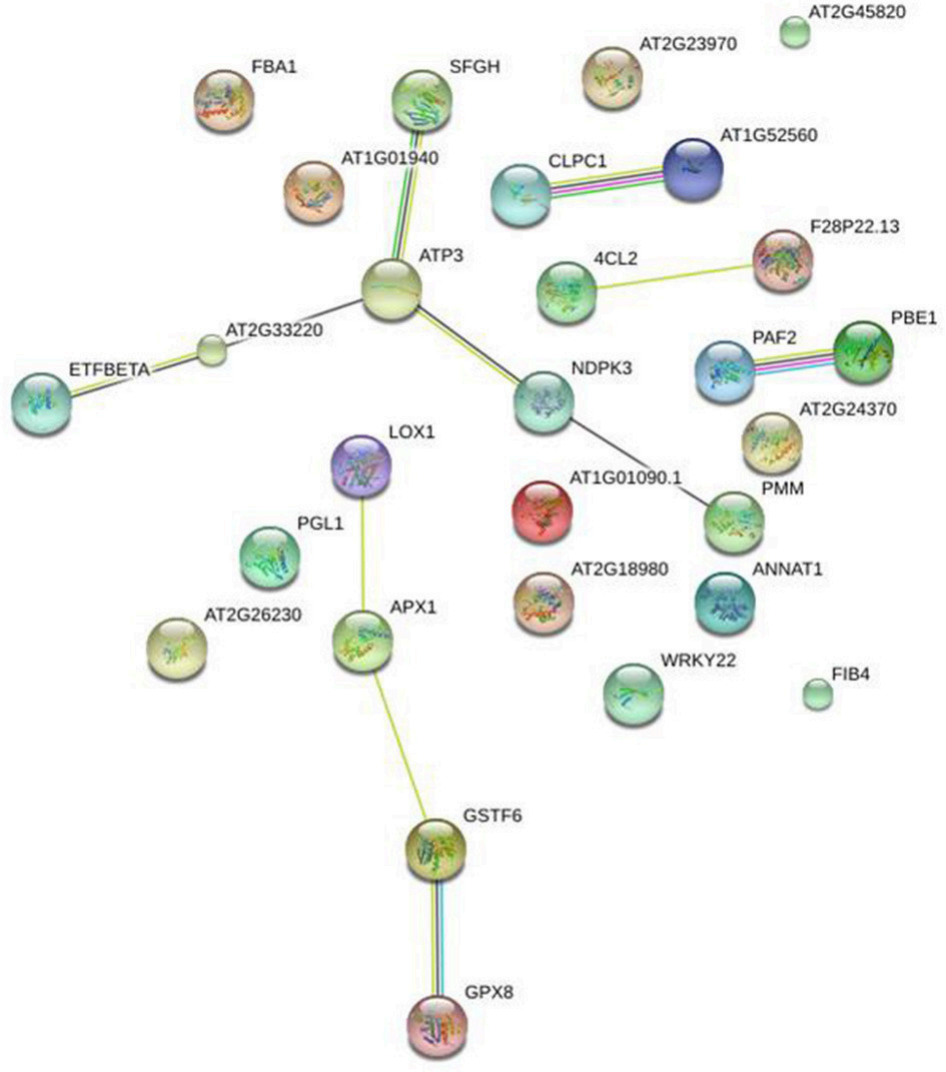
**Global protein network analysis of significantly altered peach skin proteins**. All the differentially expressed proteins were submitted to the STRING tool (version 10) (http://string.embl.de/) to predict protein–protein interaction network. Different line colors represent the types of evidence used in predicting the associations: Gene fusion (red), neighborhood (green), co-occurrence across genomes (blue), co-expression (black), experimental (purple), association in curated databases (light blue), or co-mentioned in PubMed abstracts (yellow). *Arabidopsis thaliana* and confidence level of 0.4 were used for the analysis. Details of each protein are summarized in Table 1.

STRING tool (version 10) (Szklarczyk et al., 2011) was used in order to obtain additional protein information for subsequent functional validation of the identified proteins. The software is directed to a database of known and predicted protein—protein interactions, which could be direct (physical) or indirect (functional) associations. The database retrieved such information from four major sources, including genomic information, previous experiments, co-expression, and existing knowledge (http://string.embl.de/). Interestingly, using this tool in an unbiased manner (without any additional inputs except only for protein identification number or gene symbol), the data analysis depicted that plenty of protein—protein interactions occurred in our study model as presented at Figure 7.

### The physiological function of altitude-affected skin proteins in peach fruit ripening behavior

Fruit cells employ a suite of complex mechanisms to activate acclimation processes in response to environmental factors that can be functionally categorized based on their recognition and signaling events (Molassiotis et al., 2013). An important point of the current proteomic analysis was the wide-spread up-regulation of protein abundance in response to low-altitude environment. Indeed, it was observed that 32 proteins were up-regulated at the low altitude region (Table 1), indicating that peach fruit is able to adapt the proteomic dynamic to environmental conditions, thus leading to phenotypic changes (Figure 1A). The oxidative stress is one of the major signaling pathways utilized by skin tissue to transduce extracellular stimuli into intracellular responses at the onset of fruit ripening (Pilati et al., 2014). The accumulation of glutathione-ascorbate cycle-associated enzymes, such as ascorbate peroxidase, peroxidase, and glutathione in peach grown at low altitude orchard could be associated with the elevated level of ROS, justifying the role of oxidative stress in peach fruit ripening (Prinsi et al., 2011; Nilo et al., 2012).

The fact that the antioxidant proteins were down-regulated at high altitude environment led us to hypothesize that this growing environment resulted in minimal requirement of such proteins. Antioxidant-defense proteins might be down-regulated due to the general induction of the non-enzymatic antioxidants (Figures 2A–D) and the stimulation of pigments (Figures 2E–G) that possess anti-oxidative activity (Butelli et al., 2008). Consistent with this, proteins involved in defense, such as plastid-lipid associated protein (fibrillin family protein), adenine nucleotide alpha hydrolase, annexin, agglutinin, and *S*-formylglutathione hydrolase were exclusively up-regulated at low altitude (Table 1), thus emphasizing the relevance of skin tissue as a physical barrier exerting an important part in peach fruit protection. The accumulation of plastid-lipid associated protein seems to be a ripening regulatory mechanism at low altitude grown fruits, as this protein is involved in carotenoid storage into fruit chromoplasts and induced upon high light (Rey et al., 2000), as obviously occurs at low altitude. The up-regulation of annexin (Table 1) in low altitude-grown peach fruits is in agreement with the observation that this protein act as sensor for heat-stress responses in plant cells (Wang et al., 2015). Alternatively, annexin could serve as a signal to regulate ripening or to be involved in exocytosis of cell wall-degrading enzymes, acting to sequester Ca^2+^ released from the degrading cell wall matrix, as previously proposed in strawberry and kiwifruit (Bianco et al., 2009; Tanou et al., 2015). Particularly relevant is also the up-regulation of *S*-formylglutathione hydrolase (FGH) in fruit challenged by low altitude (Table 1). FGH catalyzes the glutathione-dependent formaldehyde oxidation to formic acid which is then converted to carbonic acid or enters one-carbon cycle. Formaldehyde is a toxic compound produced in one-carbon cycle metabolism and can also arise from methanol oxidation during pectin demethylation (Gharechahi et al., 2015). Therefore, FGH might be involved in the acclimation of oxidative stress situation during peach ripening at low altitude conditions.

Fruit have developed effective acclimation mechanisms to cope with environmental conditions encountered at almost every stage of their development (Molassiotis et al., 2013). Peach fruit grown during the summer at low altitudes require more energy to adapt in high temperature (Reig et al., 2015). We observed that the abundance of various energy-related proteins was increased at low altitude environment. In particular, the up-regulation of fructose bisphosphate aldolase, pyruvate dehydrogenase and 6-phosphogluconolactonase at low altitude area (Table 1) suggested that the interaction among these proteins exerts a positive effect on the glycolytic, TCA cycle and pentose–phosphate pathways, inducing energy generation in fruit cell (Tanou et al., 2015). In addition to these energy-related enzymes, several electron transfer-associated proteins were increased at low-altitude environment. More specifically, the increased accumulation of NADH dehydrogenase (ubiquinone) and electron transfer flavoprotein subunit beta—a specific electron acceptor for dehydrogenases—along with the up-regulation of ATP synthase subunit gamma (Table 1) suggests that it would promote respiration burst and catabolic metabolism, such as redox regulation and energy accumulation in peach fruit.

Evidence defines the deep changes in sugar metabolism as a regulatory system for peach fruit ripening (Prinsi et al., 2011; Nilo et al., 2012; Desnoues et al., 2016). Among the over-expressed proteins at low altitude, the phosphomannomutase (PMM) was identified (Table 1). PMM catalyzes the interconversion between mannose-6-phosphate and mannose-1-phosphate that required for the synthesis of GDP-mannose, an intermediate in the biosynthesis of major cell wall polysaccharides (Mabeau and Kloareg, 1987) and also in ascorbate biosynthesis (Qian et al., 2007). An increase in the synthesis of precursors of cell wall polysaccharides, which are known to directly involved in fruit ripening (Tanou et al., 2015), might provide a mechanism to increase the buffering capacity of skin cell wall. Also, an induction in ascorbate synthesis is consistent with the need for the action of ascorbate-glutathione cycle at low altitude-exposed peach, as discussed above. Meanwhile, the induction of rhamnose biosynthetic enzyme at low altitude (Table 1) probably indicates that the skin tissue during ripening exhibits a loss in its capacity to convert the newly synthesized polyuronides to a more tightly bound form compared to high-altitude environment.

Two proteins were identified in skin as heat shock proteins (HSPs), i.e., a HSP20 and a small HSP (18.5 kDa class I heat shock protein). The induction of these HSPs (also called “molecular chaperones”) along with the up-regulation of peptidyl-prolyl cis-trans isomerase (Table 1), that also act as chaperone (Ou et al., 2001), is interesting because it was suggested that chaperones act as shields protecting proteins against oxidative damage, notably during peach fruit ripening (Nilo et al., 2010). In addition, it is thought that chaperones closely interact with misfolded or damaged proteins to assist their refolding upon environmental stress (Magi et al., 2004). Protein degradation plays an important role in maintaining cellular process by removing misfolded or damaged proteins and controlling the level of certain regulatory. Two proteins (proteasome subunit alpha and proteasome subunit beta) involved in ubiquitin proteasome system were identified in this study, which were both largely induced by low altitude (Table 1). Plant cells use the proteasome pathway to effectively alter their proteome so as to ensure developmental plasticity and environmental adaptation (Stone and Callis, 2007). There is evidence for involvement of the ubiquitin pathway in stress responses and fruit ripening (Molassiotis et al., 2013). Proteasome-related proteins probably regulate hormone signal transduction pathways, such as ethylene, abscisic acid (ABA) and gibberellins (GA) (Stone and Callis, 2007), thus altering the overall ripening profile.

Several other proteins were identified as low altitude-responsive proteins, including cinnamyl alcohol dehydrogenase (CAD), sinapyl alcohol dehydrogenase (SAD), and remorin (Table 1). Both CAD and SAD catalyze the monolignol biosynthesis and contribute to lignin formation. Thus, it seems likely that the up-regulation of CAD and SAD along with an increased accumulation in peroxidase may function to fruit skin against disease and herbivory. Lignin pathway is induced during stress and pathogen attack and/or defense (Dixon and Paiva, 1995) functions to enhance tissue rigidity, decreases digestibility and produce anti-microbial compounds in peach fruit at the ripe stage (Dardick et al., 2010). On the other hand, remorin belongs to a superfamily of plant-specific plasma membrane/lipid raft-associated filamentous proteins; this led to the suggestion that remorin may be associated with membrane skeletons, i.e., in superstructures that help to determine cell integrity and/or to act as scaffolds for signaling in defense or development (Bariola et al., 2004). Recently, an increase in remorin was also observed in kiwifruit experience ripening (Tanou et al., 2015; Ainalidou et al., 2016; Minas et al., 2016). Based on these results, we propose that remorin may play a more general role in macromolecular trafficking during fruit ripening. It should be noted that several of the presently detected peach skin proteins have not been previously identified in other fruit studies. For example, we observed a strong up-regulation of extradiol ring-cleavage dioxygenase at high altitude (Table 1). Dioxygenases catalyze the incorporation of both atoms of molecular oxygen into substrates using a variety of reaction mechanisms. Cleavage of aromatic rings is one of the most important functions of dioxygenases, which play key roles in the degradation of aromatic compounds. Further studies are necessary to clarify the physiological meaning of these proteins in fruit developmental process.

The current proteomic analysis revealed that the abundance of six proteins was increased in response to high altitude (Table 1); these features strongly candidate these proteins as key elements in fruit ripening at high altitude and encourage further studies to identify their downstream function. Among these proteins that up-regulated at the higher altitude, two proteins act as chaperones (chaperone ClpC and chaperone ClpB) and their up-regulation has been suggested to regulate fruit biology (Minas et al., 2012), as these proteins play pivotal role in the degradation of damaged or misfolded peptides occuring during climacteric fruit ripening (Faurobert et al., 2007). Pyruvate dehydrogenase E1 is the first component of pyruvate dehydrogenase complex (PDC). Pyruvate dehydrogenase links the glycolytic pathway to the citric acid cycle, which releases energy via NADH. In this study, pyruvate dehydrogenase E1 component subunit alpha was up-regulated at high altitude (Table 1). Given that no direct evidence has been reported that this enzyme is regulated by environmental factors in plants, we suggest that the up-regulated pyruvate dehydrogenase was beneficial for peach fruit by releasing energy during ripening, as recently suggested in harvested banana fruit (Li et al., 2015). An interesting finding in this work is the activation of TCP domain class transcription factor by high altitude (Table 1). Because TCP transcription factors can integrate hormonal, environmental and developmental signals to modulate numerous biological processes (Li, 2015), it is possible that high altitude environmental, through manipulation of TCP transcription factor, could globally regulate development programs, thereby enabling peach skin to achieve maximum fitness under these conditions. Finally, the increased abundance of lipoxygenase (Table 1), which is involved in the synthesis of oxylipins, could be associated to membrane galactolipid peroxidation and aromatic volatiles (Pilati et al., 2014), confirming that LOX could serve as biomarker of skin physiological status.

## Conclusion

This work gives, for the first time, insights to the peach skin proteome during fruit ripening at different growing environments and focus on some interesting traits of this tissue, which is significantly related with the esthetic appearance of the fruit and consumer acceptance. In this view, we observed the high altitude-related induction of the pigments and antioxidant potential. These phenotypical and physiological variations were accompanied by a general depression in proteins of defense, energy, and primary metabolism, such as glutathione-ascorbate-associated proteins, heat shock proteins, plastid-lipid associated protein, *S*-formylglutathione hydrolase, NADH dehydrogenase, and phosphoman-nomutase. However, the complex and extensive interactions of various environment factors make it difficult to define the effect of single factor on peach skin physiology. Further studies from long-term field observations are needed to deeper understand peach skin metabolism and its regulation by the environment during fruit ripening. Collectively, these findings provide the basis to understand the regulation of peach skin biology that help breeding programs aimed at improving peach quality traits.

## Author contributions

AM, IM, and GT designed the research. EK, GT, MS, IM, MM, and AM performed experiments and analyzed data. EK, GT, MS, GD, IM, MM, and AM wrote and edited the manuscript. All authors have read and approved the final manuscript.

### Conflict of interest statement

The authors declare that the research was conducted in the absence of any commercial or financial relationships that could be construed as a potential conflict of interest.

## Supplementary material

Supplementary Figure S1 **Daily climate record (low/high temperature and relative humidity) in two altitude regions**.

Supplementary Figure S2 **Subcellular localization of the identified peach skin fruit proteins**.

Supplementary Figure S3 **Mascot results for peptidyl-prolyl cis-trans isomerase (protein spot 8033) identifications based on one peptide sequences**.

Supplementary Table S1 **Quantitative data for peach skin proteins spot volumes from 2DE-gels**.

Supplementary Table S2 **Identified peach skin proteins blasted against the TAIR database and their STRING 9.0 ID**.

Supplementary Table S3 **Identification data of peach skin proteins**.

Supplementary Table S4 **Pearson's correlation coefficients calculated between fruit quality trait and proteomic characteristics**.

## References

[B1] Ainalidou A. Tanou G. Belghazi M. Samiotaki M. Diamantidis G. Molassiotis A.. (2016). Integrated analysis of metabolites and proteins reveal aspects of the tissue-specific function of synthetic cytokinin in kiwifruit development and ripening. J. Proteom. 143, 318–333. 10.1016/j.jprot.2016.02.013 26915585

[B2] Asami D. K. Hong Y.-J. Barrett D. M. Mitchell A. E. (2003). Processing-induced changes in total phenolics and procyanidins in clingstone peaches. J. Sci. Food Agric. 83, 56–63. 10.1002/jsfa.1275

[B3] Bariola P. A. Retelska D. Stasiak A. Kammerer R. A. Fleming A. Hijri M.. (2004). Remorins form a novel family of coiled coil-forming oligomeric and filamentous proteins associated with apical, vascular and embryonic tissues in plants. Plant Mol. Biol. 55, 579–594. 10.1007/s11103-004-1520-4 15604702

[B4] Benzie I. F. Strain J. J. (1996). The ferric reducing ability of plasma (FRAP) as a measure of “antioxidant power”: the FRAP assay. Anal. Biochem. 239, 70–76. 10.1006/abio.1996.0292 8660627

[B5] Bevan M. Bancroft I. Bent E. Love K. Goodman H. Dean C.. (1998). Analysis of 1.9 Mb of contiguous sequence from chromosome 4 of *Arabidopsis thaliana*. Nature 391, 485–488. 10.1038/35140 9461215

[B6] Bianco L. Lopez L. Scalone A. G. Di Carli M. Desiderio A. Benvenuto E.. (2009). Strawberry proteome characterization and its regulation during fruit ripening and in different genotypes. J. Proteomics 72, 586–607. 10.1016/j.jprot.2008.11.019 19135558

[B7] Bradford M. M. (1976). A rapid and sensitive method for the quantitation of microgram quantities of protein utilizing the principle of protein-dye binding. Anal. Biochem. 72, 248–254. 942051 10.1016/0003-2697(76)90527-3

[B8] Brand-Williams W. Cuvelier M. E. Berset C. (1995). Use of a free radical method to evaluate antioxidant activity. LWT Food Sci. Technol. 28, 25–30. 10.1016/S0023-6438(95)80008-5

[B9] Butelli E. Titta L. Giorgio M. Mock H. P. Matros A. Peterek S.. (2008). Enrichment of tomato fruit with health-promoting anthocyanins by expression of select transcription factors. Nat. Biotechnol. 26, 1301–1308. 10.1038/nbt.1506 18953354

[B10] Cantín C. M. Gogorcena Y. Moreno M. Á. (2010). Phenotypic diversity and relationships of fruit quality traits in peach and nectarine *[Prunus persica* (L.) Batsch] breeding progenies. Euphytica 171, 211 10.1007/s10681-009-0023-4

[B11] Chan Z. (2012). Proteomic responses of fruits to environmental stresses. Front. Plant Sci. 3:311. 10.3389/fpls.2012.00311 23335934 PMC3541545

[B12] Cvek J. Medić-Sarić M. Jasprica I. Zubci S. Vitali D. Mornar A.. (2007). Optimisation of an extraction procedure and chemical characterisation of Croatian propolis tinctures. Phytochem. Anal. 18, 451–459. 10.1002/pca.1001 17624905

[B13] Dardick C. D. Callahan A. M. Chiozzotto R. Schaffer R. J. Piagnani M. C. Scorza R. (2010). Stone formation in peach fruit exhibits spatial coordination of the lignin and flavonoid pathways and similarity to *Arabidopsis dehiscence*. BMC Biol. 8:13. 10.1186/1741-7007-8-13 20144217 PMC2830173

[B14] Desnoues E. Baldazzi V. Génard M. Mauroux J.-B. Lambert P. Confolent C.. (2016). Dynamic QTLs for sugars and enzyme activities provide an overview of genetic control of sugar metabolism during peach fruit development. J. Exp. Bot. 67, 3419–3431. 10.1093/jxb/erw169 27117339 PMC4892732

[B15] Deytieux C. Geny L. Lapaillerie D. Claverol S. Bonneu M. Donèche B. (2007). Proteome analysis of grape skins during ripening. J. Exp. Bot. 58, 1851–1862. 10.1093/jxb/erm049 17426054

[B16] Dixon R. A. Paiva N. L. (1995). Stress-induced phenylpropanoid metabolism. Plant Cell 7, 1085–1097. 10.1105/tpc.7.7.1085 12242399 PMC160915

[B17] Espley R. V. Hellens R. P. Putterill J. Stevenson D. E. Kutty-Amma S. Allan A. C. (2007). Red colouration in apple fruit is due to the activity of the MYB transcription factor, MdMYB10. Plant J. 49, 414–427. 10.1111/j.1365-313X.2006.02964.x 17181777 PMC1865000

[B18] Faurobert M. Mihr C. Bertin N. Pawlowski T. Negroni L. Sommerer N.. (2007). Major proteome variations associated with cherry tomato pericarp development and ripening. Plant Physiol. 143, 1327–1346. 10.1104/pp.106.092817 17208958 PMC1820912

[B19] Frett T. J. Reighard G. L. Okie W. R. Gasic K. (2014). Mapping quantitative trait loci associated with blush in peach [*Prunus persica* (L.) Batsch]. Tree Genet. Genomes 10, 367–381. 10.1007/s11295-013-0692-y

[B20] Gharechahi J. Hajirezaei M. R. Salekdeh G. H. (2015). Comparative proteomic analysis of tobacco expressing cyanobacterial flavodoxin and its wild type under drought stress. J. Plant Physiol. 175, 48–58. 10.1016/j.jplph.2014.11.001 25506766

[B21] Giraldo E. Díaz A. Corral J. M. García A. (2012). Applicability of 2-DE to assess differences in the protein profile between cold storage and not cold storage in nectarine fruits. J. Proteomics 75, 5774–5782. 10.1016/j.jprot.2012.08.005 22926270

[B22] Giusti M. M. Wrolstad R. E. (2005). Characterization and measurement of anthocyanins by UV-visible spectroscopy, in Handbook of Food Analytical Chemistry, ed Wrolstad R. E. (New York, NY: John Wiley & Sons), 19–31. 10.1002/0471709085.ch18

[B23] Goulas V. Minas I. S. Kourdoulas P. M. Lazaridou A. Molassiotis A. N. Gerothanassis I. P.. (2015). ^1^H NMR metabolic fingerprinting to probe temporal postharvest changes on qualitative attributes and phytochemical profile of sweet cherry fruit. Front. Plant Sci. 6:959. 10.3389/fpls.2015.00959 26617616 PMC4639632

[B24] Hu C. Kitts D. D. (2001). Evaluation of antioxidant activity of epigallocatechin gallate in biphasic model systems *in vitro*. Mol. Cell. Biochem. 218, 147–155. 10.1023/A:1007220928446 11330830

[B25] Hu H. Liu Y. Shi G.-L. Liu Y.-P. Wu R.-J. Yang A.-Z.. (2011). Proteomic analysis of peach endocarp and mesocarp during early fruit development. Physiol. Plant. 142, 390–406. 10.1111/j.1399-3054.2011.01479.x 21496031

[B26] Iglesias I. Echeverría G. (2009). Differential effect of cultivar and harvest date on nectarine colour, quality and consumer acceptance. Sci. Hortic. 120, 41–50. 10.1016/j.scienta.2008.09.011

[B27] Kuti J. O. (2004). Antioxidant compounds from four *Opuntia cactus* pear fruit varieties. Food Chem. 85, 527–533. 10.1016/S0308-8146(03)00184-5

[B28] Lara M. V. Borsani J. Budde C. O. Lauxmann M. A. Lombardo V. A. Murray R.. (2009). Biochemical and proteomic analysis of “Dixiland” peach fruit (*Prunus persica*) upon heat treatment. J. Exp. Bot. 60, 4315–4333. 10.1093/jxb/erp267 19734260

[B29] Leida C. Conesa A. Llácer G. Badenes M. L. Ríos G. (2012). Histone modifications and expression of DAM6 gene in peach are modulated during bud dormancy release in a cultivar-dependent manner. New Phytol. 193, 67–80. 10.1111/j.1469-8137.2011.03863.x 21899556

[B30] Li S. (2015). The *Arabidopsis thaliana* TCP transcription factors: a broadening horizon beyond development. Plant Signal. Behav. 10:e1044192. 10.1080/15592324.2015.1044192 26039357 PMC4622585

[B31] Li T. Yun Z. Zhang D. Yang C. Zhu H. Jiang Y.. (2015). Proteomic analysis of differentially expressed proteins involved in ethylene-induced chilling tolerance in harvested banana fruit. Front. Plant Sci. 6:845. 10.3389/fpls.2015.00845 26528309 PMC4606070

[B32] Lin-Wang K. Micheletti D. Palmer J. Volz R. Lozano L. Espley R.. (2011). High temperature reduces apple fruit colour via modulation of the anthocyanin regulatory complex. Plant Cell Environ. 34, 1176–1190. 10.1111/j.1365-3040.2011.02316.x 21410713

[B33] Lombardo V. A. Osorio S. Borsani J. Lauxmann M. A. Bustamante C. A. Budde C. O.. (2011). Metabolic profiling during peach fruit development and ripening reveals the metabolic networks that underpin each developmental stage. Plant Physiol. 157, 1696–1710. 10.1104/pp.111.186064 22021422 PMC3327199

[B34] Mabeau S. Kloareg B. (1987). Isolation and analysis of the cell walls of brown algae: *Fucus spiralis, F. ceranoides, F. vesiculosus, F. serratus, bifurcaria bifurcata and laminaria digitata*. J. Exp. Bot. 38, 1573–1580. 10.1093/jxb/38.9.1573

[B35] Magi B. Ettorre A. Liberatori S. Bini L. Andreassi M. Frosali S.. (2004). Selectivity of protein carbonylation in the apoptotic response to oxidative stress associated with photodynamic therapy: a cell biochemical and proteomic investigation. Cell Death Differ. 11, 842–852. 10.1038/sj.cdd.4401427 15088069

[B36] Minas I. S. Tanou G. Belghazi M. Job D. Manganaris G. A. Molassiotis A.. (2012). Physiological and proteomic approaches to address the active role of ozone in kiwifruit post-harvest ripening. J. Exp. Bot. 63, 2449–2464. 10.1093/jxb/err418 22268155 PMC3346216

[B37] Minas I. S. Tanou G. Karagiannis E. Belghazi M. Molassiotis A. (2016). Coupling of physiological and proteomic analysis to understand the ethylene- and chilling-induced kiwifruit ripening syndrome. Front. Plant Sci. 7:120. 10.3389/fpls.2016.00120 26913040 PMC4753329

[B38] Molassiotis A. Tanou G. Filippou P. Fotopoulos V. (2013). Proteomics in the fruit tree science arena: new insights into fruit defense, development, and ripening. Proteomics 13, 1871–1884. 10.1002/pmic.201200428 23986917

[B39] Montevecchi G. Vasile Simone G. Masino F. Bignami C. Antonelli A. (2012). Physical and chemical characterization of Pescabivona, a Sicilian white flesh peach cultivar [*Prunus persica* (L.) Batsch]. Food Res. Int. 45, 123–131. 10.1016/j.foodres.2011.10.019

[B40] Nilo P. R. Campos-Vargas R. Orellana A. (2012). Assessment of *Prunus persica* fruit softening using a proteomics approach. J. Proteomics 75, 1618–1638. 10.1016/j.jprot.2011.11.037 22178302

[B41] Nilo R. Saffie C. Lilley K. Baeza-Yates R. Cambiazo V. Campos-Vargas R.. (2010). Proteomic analysis of peach fruit mesocarp softening and chilling injury using difference gel electrophoresis (DIGE). BMC Genomics 11:43. 10.1186/1471-2164-11-43 20082721 PMC2822761

[B42] Ou W. B. Luo W. Park Y. D. Zhou H. M. (2001). Chaperone-like activity of peptidyl-prolyl cis-trans isomerase during creatine kinase refolding. Protein Sci. 10, 2346–2353. 10.1110/ps.23301 11604540 PMC2374073

[B43] Pilati S. Brazzale D. Guella G. Milli A. Ruberti C. Biasioli F.. (2014). The onset of grapevine berry ripening is characterized by ROS accumulation and lipoxygenase-mediated membrane peroxidation in the skin. BMC Plant Biol. 14:87. 10.1186/1471-2229-14-87 24693871 PMC4021102

[B44] Prinsi B. Negri A. S. Fedeli C. Morgutti S. Negrini N. Cocucci M.. (2011). Peach fruit ripening: a proteomic comparative analysis of the mesocarp of two cultivars with different flesh firmness at two ripening stages. Phytochemistry 72, 1251–1262. 10.1016/j.phytochem.2011.01.012 21315381

[B45] Qian W. Yu C. Qin H. Liu X. Zhang A. Johansen I. E.. (2007). Molecular and functional analysis of phosphomannomutase (PMM) from higher plants and genetic evidence for the involvement of PMM in ascorbic acid biosynthesis in *Arabidopsis* and *Nicotiana benthamiana*. Plant J. 49, 399–413. 10.1111/j.1365-313X.2006.02967.x 17217471

[B46] Re R. Pellegrini N. Proteggente A. Pannala A. Yang M. Rice-Evans C. (1999). Antioxidant activity applying an improved ABTS radical cation decolorization assay. Free Radic. Biol. Med. 26, 1231–1237. 10.1016/S0891-5849(98)00315-3 10381194

[B47] Reig G. Alegre S. Gatius F. Iglesias I. (2015). Adaptability of peach cultivars [*Prunus persica* (L.) Batsch] to the climatic conditions of the Ebro Valley, with special focus on fruit quality. Sci. Hortic. 190, 149–160. 10.1016/j.scienta.2015.04.019

[B48] Rey P. Gillet B. Römer S. Eymery F. Massimino J. Peltier G.. (2000). Over-expression of a pepper plastid lipid-associated protein in tobacco leads to changes in plastid ultrastructure and plant development upon stress. Plant J. 21, 483–494. 10.1046/j.1365-313X.2000.00699.x 10758499

[B49] Stone S. L. Callis J. (2007). Ubiquitin ligases mediate growth and development by promoting protein death. Curr. Opin. Plant Biol. 10, 624–632. 10.1016/j.pbi.2007.07.010 17851112

[B50] Szklarczyk D. Franceschini A. Kuhn M. Simonovic M. Roth A. Minguez P.. (2011). The STRING database in 2011: functional interaction networks of proteins, globally integrated and scored. Nucleic Acids Res. 39, 561–568. 10.1093/nar/gkq973 21045058 PMC3013807

[B51] Tadiello A. Ziosi V. Negri A. S. Noferini M. Fiori G. Busatto N.. (2016). On the role of ethylene, auxin and a GOLVEN-like peptide hormone in the regulation of peach ripening. BMC Plant Biol. 16:44. 10.1186/s12870-016-0730-7 26863869 PMC4750175

[B52] Tanou G. Job C. Belghazi M. Molassiotis A. Diamantidis G. Job D. (2010). Proteomic signatures uncover hydrogen peroxide and nitric oxide cross-talk signaling network in Citrus plants. J. Proteome Res. 9, 5994–6006. 10.1021/pr100782h 20825250

[B53] Tanou G. Job C. Rajjou L. Arc E. Belghazi M. Diamantidis G.. (2009). Proteomics reveals the overlapping roles of hydrogen peroxide and nitric oxide in the acclimation of citrus plants to salinity. Plant J. 60, 795–804. 10.1111/j.1365-313X.2009.04000.x 19682288

[B54] Tanou G. Minas I. S. Karagiannis E. Tsikou D. Audebert S. Papadopoulou K. K.. (2015). The impact of sodium nitroprusside and ozone in kiwifruit ripening physiology: a combined gene and protein expression profiling approach. Ann. Bot. 116, 649–662. 10.1093/aob/mcv107649-662 26159933 PMC4578001

[B55] Trainotti L. Bonghi C. Ziliotto F. Zanin D. Rasori A. Casadoro G. (2006). The use of microarray μPEACH1.0 to investigate transcriptome changes during transition from pre-climacteric to climacteric phase in peach fruit. Plant Sci. 170, 606–613. 10.1016/j.plantsci.2005.10.015

[B56] Tuan P. A. Bai S. Yaegaki H. Tamura T. Hihara S. Moriguchi T.. (2015). The crucial role of PpMYB10.1 in anthocyanin accumulation in peach and relationships between its allelic type and skin color phenotype. BMC Plant Biol. 15:280. 10.1186/s12870-015-0664-5 26582106 PMC4652394

[B57] Wang L. Zhao S. Gu C. Zhou Y. Zhou H. Ma J.. (2013). Deep RNA-Seq uncovers the peach transcriptome landscape. Plant Mol. Biol. 83, 365–377. 10.1007/s11103-013-0093-5 23783411

[B58] Wang X. Ma X. Wang H. Li B. Clark G. Guo Y.. (2015). Proteomic study of microsomal proteins reveals a key role for Arabidopsis annexin 1 in mediating heat stress-induced ncrease in intracellular calcium levels. Mol. Cell. Proteomics 14, 686–694. 10.1074/mcp.M114.042697 25587034 PMC4349987

[B59] Wolfe K. Wu X. Liu R. H. (2003). Antioxidant activity of apple peels. J. Agric. Food Chem. 51, 609–614. 10.1021/jf020782a 12537430

[B60] Zhang L. Yu Z. Jiang L. Jiang J. Luo H. Fu L. (2011). Effect of post-harvest heat treatment on proteome change of peach fruit during ripening. J. Proteomics 74, 1135–1149. 10.1016/j.jprot.2011.04.012 21550427

[B61] Ziliotto F. Begheldo M. Rasori A. Bonghi C. Tonutti B. (2008). Transcriptome profiling of ripening nectarine (*Prunus persica* L. Batsch) fruit treated with 1-MCP. J. Exp. Bot. 59, 2781–2791. 10.1093/jxb/ern136 18515268 PMC2486471

[B62] Ziogas V. Tanou G. Molassiotis A. Diamantidis G. Vasilakakis M. (2010). Antioxidant and free radical-scavenging activities of phenolic extracts of olive fruits. Food Chem. 120, 1097–1103. 10.1016/j.foodchem.2009.11.058

