# Supplementary material for: Comparative Physiological and Proteomic Analysis Reveal Distinct Regulation of Peach Skin Quality Traits by Altitude
Source: Front Plant Sci. 2016 Nov 10;7:1689. doi: 10.3389/fpls.2016.01689 (PMC5102882; doi:10.3389/fpls.2016.01689)

Supplementary Figure S1. Daily climate record (max/min temperature and relative humidity) in high- and low-altitude regions. Colored boxes indicate the harvest period in each region.

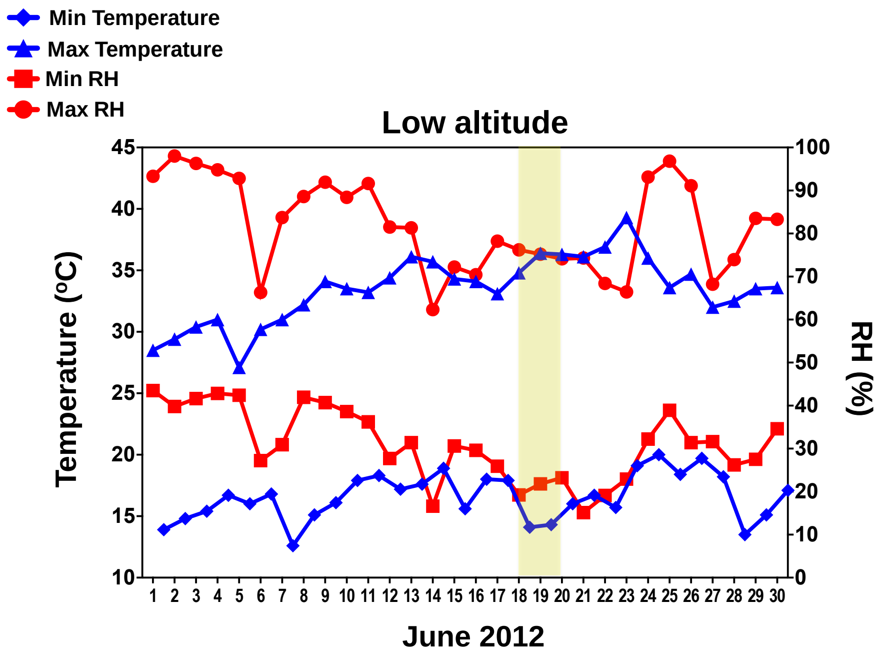

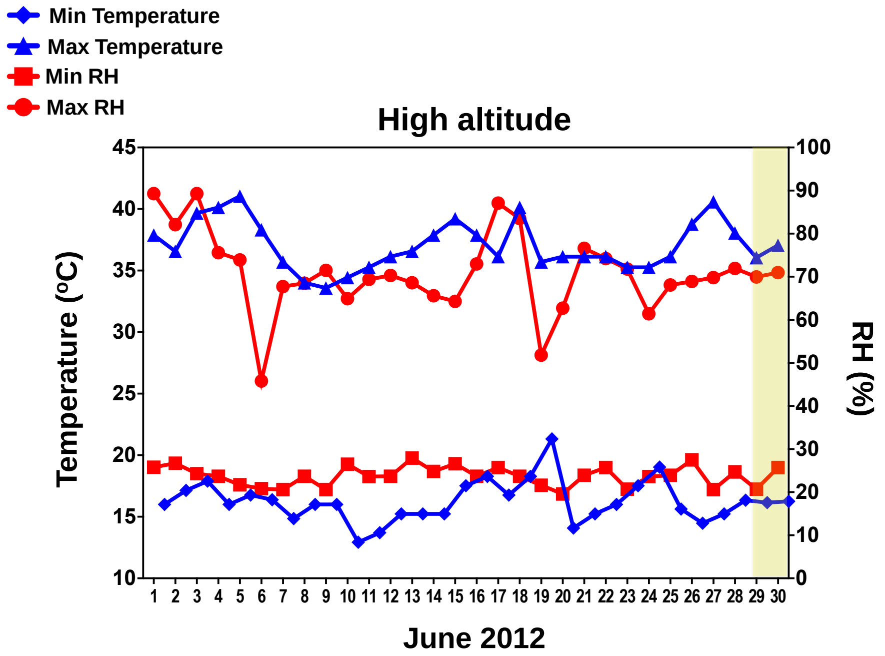

Supplement: Supplementary Figure S1 — Daily climate record (low/high temperature and relative humidity) in two altitude regions. [file DataSheet1.PDF]
