# Supplementary material for: Comparative Physiological and Proteomic Analysis Reveal Distinct Regulation of Peach Skin Quality Traits by Altitude
Source: Front Plant Sci. 2016 Nov 10;7:1689. doi: 10.3389/fpls.2016.01689 (PMC5102882; doi:10.3389/fpls.2016.01689)
Supplement: Supplementary Figure S2 — Subcellular localization of the identified peach skin fruit proteins. [file DataSheet2.PDF]

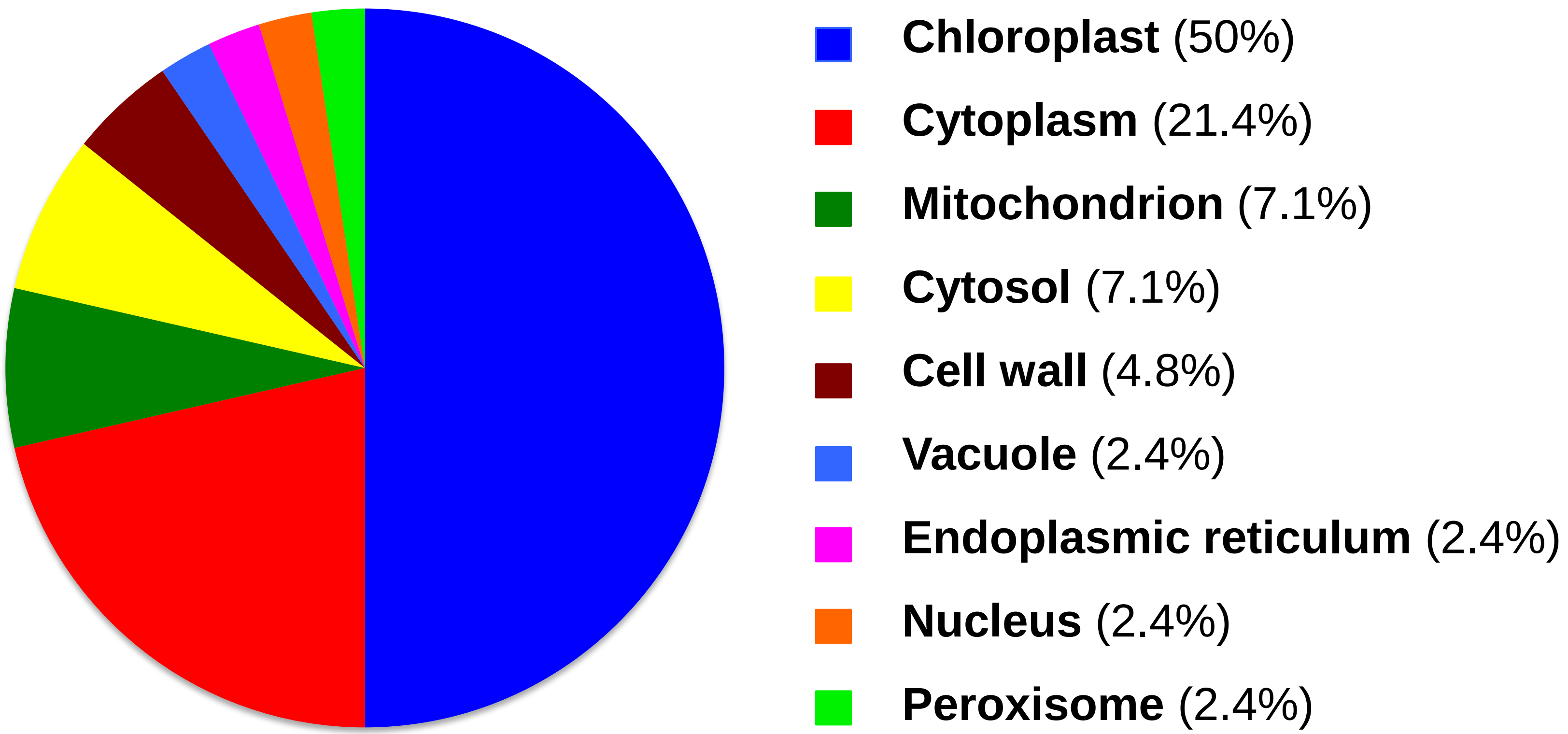
Chloroplast (50%)
Cytoplasm (21.4%)
Mitochondrion (7.1%)
Cytosol (7.1%)
Cell wall (4.8%)
Vacuole (2.4%)
Endoplasmic reticulum (2.4%)
Nucleus (2.4%)
Peroxisome (2.4%)
