# Supplementary material for: Comparative Physiological and Proteomic Analysis Reveal Distinct Regulation of Peach Skin Quality Traits by Altitude
Source: Front Plant Sci. 2016 Nov 10;7:1689. doi: 10.3389/fpls.2016.01689 (PMC5102882; doi:10.3389/fpls.2016.01689)
Supplement: Supplementary Figure S3 — Mascot results for peptidyl-prolyl cis-trans isomerase (protein spot 8033) identifications based on one peptide sequences. [file DataSheet3.PDF]

Peptide Identification Details

Options

Load | Save | Factory Defaults

Display Options

Charge Detail Level:

- (•) All
- ( ) +1; +2

Annotation Threshold:

- (•) 5.0 % of base peak
- ( ) 1000 absolute

- [x] Show legend
- [ ] Show reference spectrum

- [ ] Use search settings

Match Tolerances

Mass analyzer: ITMS

Match tolerance: 0.8 Da

Fragments

Activation Type: CID

Ion Series

- [ ] a
- [ ] x
- [x] b
- [x] y
- [ ] c-1
- [ ] z
- [ ] c
- [ ] z+1
- [ ] c+1
- [ ] z+2

Neutral Losses

- [x] $-H_2O$
- [x] $-NH_3$

PTMs

- [x] $-H_3PO_4$ (-P)

Other

- [ ] Immonium
- [x] Precursor

Peptide Summary

Sequence: TSRPVVVADCGQLS, C10-Carbamidomethyl (57.02146 Da)
Charge: +2, Monoisotopic m/z: 744.87769 Da (+0.26 mmu/+0.35 ppm), MH+: 1488.74809 Da, RT: 34.86 min,
Identified with: Sequest HT (v1.3); XCorr:2.19, Ions matched by search engine: 0/0
Fragment match tolerance used for search: 0.6 Da

Fragment Matches

Value Type: Theo. Mass [Da]

Ion Series | Neutral Losses | Precursor Ions

#1	b^+	b^{2+}	Seq.	y^+	y^{2+}	#2
1	102.05496	51.53112	T			14
2	189.08699	95.04713	S	1387.69990	694.35359	13
3	345.18811	173.09769	R	1300.66787	650.83757	12
4	442.24088	221.62408	P	1144.56675	572.78701	11
5	541.30930	271.15829	V	1047.51398	524.26063	10
6	640.37772	320.69250	V	948.44556	474.72642	9
7	739.44614	370.22671	V	849.37714	425.19221	8
8	810.48326	405.74527	A	750.30872	375.65800	7
9	925.51021	463.25874	D	679.27160	340.13944	6
10	1085.54086	543.27407	C-Carbami...	564.24465	282.62596	5
11	1142.56233	571.78480	G	404.21399	202.61063	4
12	1270.62091	635.81409	Q	347.19252	174.09990	3
13	1383.70498	692.35613	L	219.13394	110.07061	2
14			S	106.04987	53.52857	1

Fragment Spectrum

Extracted from: G:\Orbitrap_Backup\January_2014\25011438.raw #872 RT: 34.86
ITMS, CID@35.00, z=+2, Mono m/z=744.87769 Da, MH+=1488.74809 Da, Match Tol.=0.8 Da

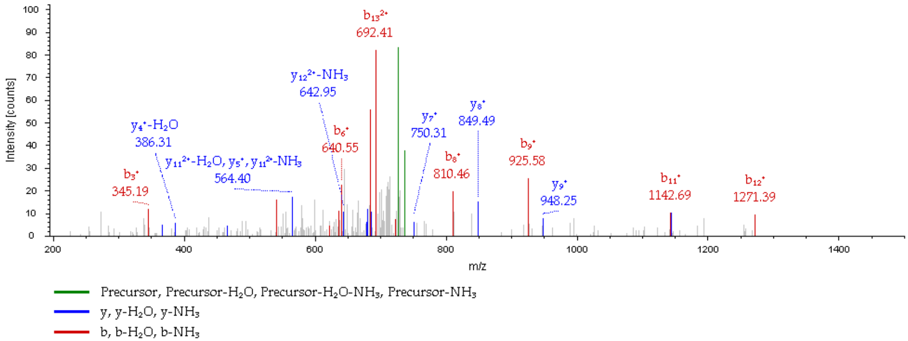

OK | Help
